# Literary Notes

**Published:** 1897-09

**Authors:** 


### Literary Notes.

THE *Georgia Journal of Medicine and Surgery* made its bow to the professional public, July 15, 1897. It is edited by Drs. St. J. B. Graham, D. E. Dudley, and is published at Savannah, Ga. While we bespeak success to our new contemporary, we doubt the propriety of its publishing what it calls its consultation columns. Medical journals that have heretofore pursued a similar policy have not risen to the high plane to which our Georgia friend aspires and to which its long list of honorable collaborators would seem to give it appropriate place.

THE tenth annual report of the managers of the St. Lawrence State Hospital has been issued. It covers the period for the year ending September 30, 1896, during which there were 157 men and 133 women admitted as patients. There were 155 men and 120 women discharged and died during the year and a total of 1,268 patients remained in the hospital under care and treatment. The superintendent, Dr. Peter M. Wise, is well known in Buffalo as a competent alienist and an administrative officer of ability. The successful management of the hospital is assured during his incumbency.

THE NEEDS AND RIGHTS OF OLD AGE is the title of a doctorate address delivered at the commencement exercises of the hospital medical college, at Louisville, Ky., July 1, 1897, by Prof. Isaac Newton Love, M. D., of St. Louis. The occasion was a happy one, the wealth and fashion of Louisville were assembled at McCauley's theatre and Professor Love was at his best. This scholarly address, that abounds in classic prose and poetic lore, will hereafter be quoted as one of the best doctorate addresses of the season. It was a graceful act of the Louisville college to invite Dr. Love to be the guest of honor at its commencement and the learned St. Louisian returned the courtesy with interest.

---

THE Cincinnati hospital has issued its twenty-sixth annual report to the mayor of Cincinnati, which embraces the fiscal year ending December 31, 1896. The hospital buildings occupy an entire square, being 448 feet from north to south and 340 feet from east to west. The number of patients treated during the year, exclusive of the receiving ward, was 5,964 and the appropriation by the city for the expenses of the hospital was $114,000.40. It is doubtful if the same number of patients have been cared for at as small an expenditure anywhere in the country. The advantages growing out of a large general hospital supported by the city treasury are manifold, and the example of Cincinnati is worthy of imitation—if not emulation.

